# Auto-generating databases of Yield Strength and Grain Size using ChemDataExtractor

**DOI:** 10.1038/s41597-022-01301-w

**Published:** 2022-06-09

**Authors:** Pankaj Kumar, Saurabh Kabra, Jacqueline M. Cole

**Affiliations:** 1 Cavendish Laboratory, Department of Physics, University of Cambridge, J.J. Thomson Avenue, Cambridge, CB3 0HE UK; 2 ISIS Neutron and Muon Source, STFC Rutherford Appleton Laboratory, Didcot, OX11 0QX UK

**Keywords:** Mechanical engineering, Metals and alloys, Mechanical properties

## Abstract

The emerging field of material-based data science requires information-rich databases to generate useful results which are currently sparse in the stress engineering domain. To this end, this study uses the’materials-aware’ text-mining toolkit, ChemDataExtractor, to auto-generate databases of yield-strength and grain-size values by extracting such information from the literature. The precision of the extracted data is 83.0% for yield strength and 78.8% for grain size. The automatically-extracted data were organised into four databases: a Yield Strength, Grain Size, Engineering-Ready Yield Strength and Combined database. For further validation of the databases, the Combined database was used to plot the Hall-Petch relationship for, the alloy, AZ31, and similar results to the literature were found, demonstrating how one can make use of these automatically-extracted datasets.

## Abstract (summary)

**Table:** 

Measurement(s)	yield strength • grain size
Technology Type(s)	natural language processing

## Background & Summary

Engineering materials and their design are of great importance in most major industries, such as the manufacturing, transportation, aeronautics, and sustainable energy sectors. The stress-strain behaviour^1^ of these materials is both fundamental to their performance and key to modelling other advanced properties. Improvements to the workflow of material analysis and design are critical to reducing’material-to-market’ timescales and enhancing the quality of results. Traditionally, the experimental methods involved in discovering and understanding new materials rely on trial-and-error and can be inefficient^2^. However, there has been a recent trend to use new data-driven techniques to aid in material science research^3^.

The success of data-driven research is highly dependent on the quality and availability of material databases. This has been highlighted by recent efforts, such as the Materials Genome Initiative^4^, which aims to incorporate’big data’ into the material development workflow. While projects that branch from this initiative have been successful in generating large material databases (e.g., Materials Project^5^), these do not include stress-strain information that would be useful to an engineer. Alternatively, platforms such as, Citrination^6^ and MaterialDataFacility^7^ (MDF), aim to centralise material data by providing a space for researchers to upload and share their own results. These can be downloaded to a local machine or accessed via an Application Programming Interface (API) which permit easier implementation into material data-science workflows^8^. However, such platforms rely on researchers making use of them in the first place meaning that useful material information predating the creation of the above platforms is unlikely to be provided. This, combined with the need to adhere to different data formats for the different platforms, means that there is currently a limited amount of usable data on stress-strain behaviour. Furthermore, the lack of uniformity amongst the uploaded data themselves, causes difficulties in combining data from different uploaders. Therefore, such platforms are currently best served as a material look-up tool rather than a source of information-rich databases for data-driven material research, particularly when dealing with engineering properties.

It is important to note that the problem is not the lack of data, but rather, the difficulty in collecting and packaging material data in a format that is machine readable and ready for use. Given that the understanding of engineering-material properties and the corresponding relationships are so crucial, an ever-growing literature consisting of useful material information is available. Yet, the fragmented distribution of this literature information means that it cannot be directly implemented in the current state. One must first collate all the material information into a database to which data-driven techniques can then be applied. This is a seemingly impossible task to complete manually. However, text-mining techniques can be exploited to automate the extraction of material information from the literature. In other domains, such as chemistry or biomedicine, text-mining tools have been successfully utilised for automatic information extraction^9,10^. Yet, to the best of our knowledge, no such method has been successfully applied to the engineering field with respect to the extraction of stress-strain related properties.

This work presents a material database for studying stress-engineering material properties that has been produced using automatically extracted information from the literature. While this study is primarily focused on developing a database of metals and alloys used for structural applications, the full database has a more general purpose as its content spans various domains, including soft materials whose engineering target properties have been extracted. Yield strength and grain size were chosen as the target engineering properties because they are good descriptors of the mechanical and structural properties of a material, and are also linked via the “Hall-Petch Relation”,^11,12^ which has been experimentally justified in the micrometre range. Yield strength is the stress at which the material behaviour fundamentally changes from elastic to plastic; it is a key property in defining the stress-strain behaviour of materials. ‘Grain size’ is a microstructural feature and is defined as the length scale of similarly oriented crystalline units. Such information was automatically extracted from a corpus of papers that were downloaded from the engineering literature in a high-throughput fashion. Their contents were mined using a bespoke version of ChemDataExtractor^13,14^, a state-of-the-art materials-aware natural language processing (NLP) tool, that was created specifically for this study on engineering materials. The resulting database allows for deeper study on the target properties, for example, statistical analysis on the aforementioned “Hall-Petch Relation” which has not previously been possible at such a large scale. Thus, the provided databases allow for a unique perspective and analysis on engineering-material properties.

## Methods

The entire process of producing the automatically extracted material databases can be summarised in three major steps; first, article retrieval involves the generation of a corpus of relevant articles that are likely to contain the target properties. Second, information extraction includes the use of a bespoke version of ChemDataExtractor 2.0^14^ to read and extract material data from the corpus. The third and final step is post-processing, which filters and organises the data into machine-readable formats while simultaneously attempting to minimise errors in the database.

### Article retrieval

Article retrieval is the process of searching the literature and downloading papers that are likely to be relevant to this study. For this, webscraping tools were written in Python and were targetted primarily at the publisher Elsevier since their Text and Data-Mining (TDM) policy allow for the downloading of full-text articles in extensible markup language (XML) format from their repositories. This format is well suited for information extraction using ChemDataExtractor since it contains tags that identify document elements such as titles, abstracts, tables and paragraphs, which can be used to convert input articles to plain text. Furthermore, Elsevier provides an API specifically for article search and data mining. The written webscraping tool makes use of this API alongside the Python library *Requests* to take a search body as an input, mimic Elsevier’s web search and download the returned articles.

As the primary focus of this study is to extract yield strength, the search query used was *“yield strength” OR “yield stress”*. Here, search operators are used to ensure that either term appears as a pair of words somewhere within the text body of an article. In particular, the quotation marks enforce the pair requirement, which is necessary since querying the individual words would return irrelevant search results. For example, search results would often include papers from agriculture when the search query entered is simply *yield strength*, due to the inclusion of *yield* in the text bodies. Since this study focuses on grain size in the context of studying its relation with yield strength, no direct search for grain size was required because articles that feature grain size that would be useful for this study are most likely to be contained implicitly within those that mention yield strength or yield stress.

It should be noted that the Elsevier API is limited in that it can only handle a maximum of 6,000 articles per search request. Also, each request is static, meaning that identical search queries should yield the same list of articles. These limitations were circumvented by retrieving articles that specify the year of publication within the search request. It was also found that full text articles predating 2001 would often not be available or would cause issues in the information-extraction pipeline; however, the actual number of articles predating 2001 is relatively small. Thus, articles published between 2001 and 2021 were downloaded, as this range ensured that the maximum number of articles were downloaded within the restrictions imposed by the API.

The downloaded articles were then organised and filtered through a series of checks based on the XML tags contained within the downloaded article. First, articles were filtered out if they did not contain the full text within the downloaded XML file; this was done by checking for the XML tag: “*<xocs:rawtext>”*. Next, the document type was checked, whereby the downloads that were not research articles were filtered out; this required the tag *““<xocs:document-subtype>”* to be assigned the value *“fla”*. Finally, open-access articles were tagged for use in evaluation by checking if *“<openaccess>”* is true.

The article-retrieval process yielded 93,202 articles of which 11,175 are open access. These articles are ready to use in the information-extraction stage, which is described in the following subsection.

### Changes to ChemDataExtractor 2.0

The information-extraction stage entails reading an input article and outputting a dictionary of records that contain target properties. As previously mentioned, this makes use of a bespoke version of ChemDataExtractor 2.0 that has been appropriately modified for this study. Full details of ChemDataExtractor and its design are given by Swain *et al*.^13^ and Mavracic *et al*.^14^. The noteworthy changes for this work include additions to the Chemical Named Entity Recognition part of the ChemDataExtractor operational pipeline and new property models for Information Extraction which enable ChemDataExtractor to better extract engineering-material properties, given that the tool has not been used in this field before. The details of these changes will be discussed in the next two subsections.

#### Property models

Within an NLP pipeline, information extraction aims to construct semantic relationships using identified named entities and the given context. Most commonly, rule-based methods are employed to manually construct grammars that give meaning to named entities in the text^15^. ChemDataExtractor 2.0 uses multiple rule-based grammars, in the form of property models, that are specialised for the chosen property to be extracted. In this work, two new property models were constructed for grain size and yield strength as these properties were not originally supported by the ChemDataExtractor 2.0 tool.

In the case of grain size, ChemDataExtractor 2.0 has an inbuilt LengthModel which defines the grammar to handle extraction of the units of grain size; this parents the grain-size property model. The units and dimensions for stress-related quantities, such as yield strength, had to be created from scratch. The standard, and almost exclusively used unit for stress in the engineering literature is *Pascal* for which parsing rules had to be defined. By building upon the base unit models of ChemDataExtractor 2.0, a new unit model was constructed to support stress-related quantities (StressModel). Once the grammar to handle units had been defined, four property models were constructed to handle text and table mentions of grain size and yield strength. The code snippet below shows the property model that was defined for yield strength.

Each model has its own set of rule-based parsers which identify whether or not the context surrounding a named entity is in fact related to the desired property. When dealing with tabular data, the AutoTableParser, which comes packed with ChemDataExtractor 2.0, is used in both models because of its high accuracy’ out of the box’. Grain size and yield strength values were found to be reported with prefixes that are mostly the same within the engineering literature. Therefore, the inherent *TemplateParsers* were extended for each property, meaning that additional rules were written to detect the desired property. Both single and multiple instances of a property’s value were considered and used to construct the parsing rules which were applied to sentences to extract information that is structured as described in the property models. Using a mixture of manually constructed sentences and ones found in literature as test sentences, the parsing rules were extended to handle previously unsupported cases. These rules are included in the new *YieldStrengthParser* and *MultiYieldStrengthParser* (and similarly for grain size) for clarity purposes; however, these can be used for any property model.

#### Chemical Named Entity Recognition (CNER)

ChemDataExtractor employs a hybrid approach to Chemical Named Entity Recognition (CNER); machine-learnt, dictionary-based and rule-based methods are all used. Here, the rule-based and dictionary-based methods were extended to be better suited for our targetted domain and properties. Firstly, existing parsing rules were modified to identify representations of alloys that are commonly found in the engineering literature. For example, alloys Na-3at.%W-1.7at.%Fe, Mg–2.7Nd–0.6Zn–0.5Zr and others of similar form were not recognised, or only a part of the compound was extracted, in the original version of ChemDataExtractor 2.0. Thus, new rules were implemented that made use of regular expressions to correctly identify alloys of this kind. An example of a newly implemented rule is given below.

Here, element_regex is a set of regular-expression rules that are designed to match an element from the periodic table; the + operator considers the next token, allowing for the rule to recognise alloy names that span over multiple characters; OneOrMore matches the following expression at least once; R begins a regular-expression environment.

The engineering domain also makes extensive use of trade names in place of chemical compositions when describing material properties such as AZ31 or Waspaloy. Trade names are commercialised materials that have specific compositions. Thus, a dictionary of common material trade names was constructed and used in the CNER stage. This dictionary was constantly updated as new names were found.

The priority of CNER methods were adjusted such that the purpose-built rules were employed first. Thereby, highest priority is given to the trade name dictionary, then the rule-based alloy detection as described above, and then the default CNER methods that are included in ChemDataExtractor 2.0.

Modifications to the handling of certain symbols were also made. Symbols such as “-” or “*μ*” can be represented by multiple unicode characters that look identical. This is problematic as different journals may use different unicode characters to represent the same thing and, if this is not accounted for, ChemDataExtractor will not be able to extract these correctly. For example, a common unit for grain size is “µm”, where the symbol “µ” means “micro”. There are two characters in unicode which look almost identical, the symbol for micro µ, and the Greek letter mu, *μ*. The same is true for hyphens as there are multiple possible unicode characters that can be used. Thus, these extra characters were explicitly included in the parsing rules for units and chemical entities in order to ensure the correct extraction.

### Information extraction

For the information-extraction stage, ChemDataExtractor is used to process the input articles and parse the text to extract the target properties. This was carried out on the high-performance computing cluster Cooley at the Argonne Leadership Computing Facility.

#### Document processing

The *reader* package provided by ChemDataExtractor was utilised to convert the downloaded XML articles into standardised plain text, which was then converted into a Python *Document* class designed for easier handling and to be used for information extraction. Using the XML tags, document elements such as title, abstract, heading, paragraph, table, etc. can be identified. These elements are extracted as plain text and form a *Document* object to which the property-model parsers will be assigned to extract the relevant properties. During document processing, useful article information such as DOI, author and title, are compiled as metadata which are assigned to the *Document* object for that article. These metadata are appended to any extracted record in the subsequent step of the information-extraction pipeline.

#### Extraction of properties from documents

Once a *Document* object has been created, the appropriate property models are assigned depending on the element from which information is to be extracted. Tabular and textual data are handled with different models as they require different parsing rules. Whenever a table element is to be parsed, *TableYieldStrength* and *TableGrainSize* property models are assigned to the *Document* whereas text elements make use of *YieldStrength* and *GrainSize* models.

**Fig. 1:**
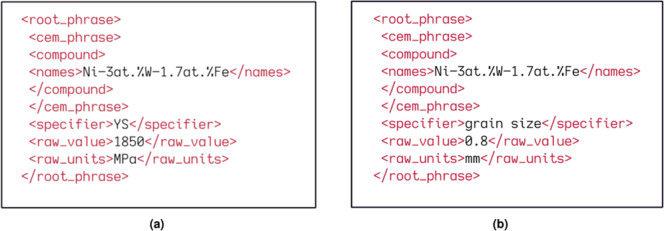
The xpath trees interpreted with the parsing rules for: (**a**) textual Yield Strength and (**b**) textual Grain Size applied to the example sentence: *Ni–3at.%W–1.7at.%Fe (YS = 1850 MPa) has an average grain size is 0.8 mm*.

If a parser identifies a value based on the defined rules, an xpath tree is returned which contains the extracted information. For example, Fig. 1 shows the result of parsing using the property models for text grain size and yield strength on the following sentence: *Ni–3at.%W–1.7at.%Fe (YS = 1850 MPa) has an average grain size is 0.8 mm*. The extracted value and units are normalised by ChemDataExtractor before being converted into a Python dictionary. Additional information including the property model, parser used and article metadata are appended to the dictionary that contains the extracted record, which is finally stored into a JSON file for further processing.

### Post processing

The raw data from ChemDataExtractor are cleaned before being organised into databases so as to increase the likelihood of correct entries. First, records that are incomplete are not stored, meaning that if any of compound, value or unit fields are empty, then they are discarded. The remaining records are imported into Python, using the library *Pandas*, as two dataframes, one for yield strength and one for grain size, with each key of the imported dictionary used as the columns of the dataframes i.e. Compound, Value, Unit etc. Next, multiple filters are applied to the Compound column of the dataframe. The first filter is the dictionary of trade names that was implemented as part of the CNER. Since these names were defined manually, they are likely to have been extracted correctly and thus they are stored directly, without verification. Next, a Materials Parser^16^ is applied to the Compound column of the remaining records in order to remove entries that do not have any elements. This parser has been designed to identify the compositions or elements or a single-string input. Here, it is employed to remove compound names that are words which are not used to name materials, such as “Nano grained”. However, the parser is very lenient and it will identify entries with compound names that have element symbols even though they are incorrect when considered in context. For example, a common error in the extracted compound name was “UFG”. The Materials Parser would identify this as a composition and keep the record within the database. Within context, “UFG” is an acronym for Ultra Fine Grained and it is therefore an incorrectly/partly extracted compound name. Furthermore, single elements would also be accepted by the Materials Parser. Although there may be studies on the mechanical properties of single elements such as titanium, they are often used to discuss the effects they have when they are added in varying quantities to other alloys and therefore, are usually a source of error since the database would sometimes include the single-element addition instead of the entire alloy composition. Problematic entries of this type were dealt with by employing a blacklist in this work. The blacklist includes compound names such as, *“HEAs”, “UFG”, “magnesium”*. Records with a compound that appears in the blacklist are tagged accordingly.

To study the link between yield strength and grain size, the two databases need to be combined. Entries with the same DOI are compared and assigned to one another if the following criteria are met: (1) The same number of entries are present in either database for the unique DOI. (2) The entries have the same compound name and DOI. Since it is generally accepted that smaller grain sizes pertain to larger yield strengths^1^, grain-size entries are inversely matched with their corresponding yield-strength entries and are copied to a new combined database. This combined database can be used for studying the Hall-Petch relationship using automatically extracted data.

All of the processed data are then exported in three formats: CSV, JSON and MongoDB. These formats have been chosen since they can be easily implemented in any system using different programming languages for further data-driven study while also supporting database look-up features.

## Data Records

Four databases have been assembled to portray the auto-extracted data in distinct sets: a Yield Strength, Grain Size, Engineering-Ready Yield Strength and Combined Yield Strength & Grain Size database. Each of these is available to download from *Figshare*^17^ in JSON, CSV and MongoDB formats.

The data records have mutual descriptors which are shown in Table 1. *Compound* is a list of extracted compound names where each entry represents alternative forms of the same compound if found in the article during the information-extraction process. *Value* and *Units* are the extracted value and units which have been normalised as defined by the property models. In cases where more than one value is extracted, when a range of values is extracted for example, *Value* will be a list. These data are given in their original form under *Raw Value* and Raw Unit, which contains the extracted information before being normalised. Additional fields describing article metadata and parsing methods are also included.

**Table 1:** Description of data records contained in the databases.

Database Key	Description	Data Type
Compound	Extracted compound names that have been normalised. May contain more than one entry if multiple names are given to the same compound.	List of String Type
Blacklisted Compound?	Flag that identifies if the extracted compound names contain a blacklisted compound. The list of blacklisted compound is manually curated.	Boolean
Value	Extracted value that has been normalised using ChemDataExtractor	List of Float Type
Units	Extracted units that have been normalised	String Type
Raw Value	Extracted value as seen in text without any normalisation	String Type
Raw Units	Extracted units as seen in text without any normalisation	String Type
Parsing Method	Identifies if the text or table parsing method is used from extraction of the record	String Type
DOI	Unique identifier of the source article	String Type
Article Title	Title of source article	String Type
Author	Authors of source article	String Type
Journal	Journal of source article	String Type
Date	Date-of-publishing of source article	String Type
Open Access	Identifies if the source article is open-access	Boolean

The Yield Strength database contains 64,269 records of which 25,555 records come from text-parsing methods and 38,714 come from table parsing. 7,172 compounds were identified as *Blacklisted Compound* during the post-processing stage and have been flagged as such. The Grain Size database contains 30,285 records of which 23,649 were extracted using text parsing and 6,636 were extracted from tables. 5,138 compounds were blacklisted and tagged accordingly.

The Engineering-Ready Yield Strength and Combined Yield Strength & Grain Size databases are derived from the two main sets described above. The Engineering-Ready Yield Strength Database limits the yield-strength value to be between 0 MPa and 1500 MPa. This subset contains 48,430 records, of which 28,263 were extracted from text and 20,167 were extracted from tables. The Combined Yield Strength & Grain Size matches extracted yield-strength and grain-size values from the same article and contains 2,032 complete records (Compound, Blacklisted Compound? Yield Strength Value, Yield Strength Unit, Grain Size Value, Grain Size Unit, DOI, Open Access).

## Technical Validation

Technical validation is carried out in order to measure the reliability of the extracted data. Two methods are used for this task: one calculates the evaluation metrics, precision, recall and F-score which are commonly used to measure the efficacy of NLP results; the other is a statistical approach whereby the distribution of data is visualised and the Hall-Petch relation for AZ31 is plotted to compare with results from the literature.

### Evaluation Metrics

The reliability of the data extracted can be measured by the evaluation metrics: precision, recall and F-score. The precision describes the percentage of correct data that have been extracted. Recall is the fraction of properly extracted records over the number of possible records in a document. F-score is the harmonic mean of precision and recall. These evaluation metrics are given by:

1 $$precision=\frac{TP}{TP+FP}$$

2 $$recall=\frac{TP}{TP+FN}$$

3 $$F \mbox{-} score=2\cdot \frac{precision\cdot recall}{precision+recall}$$

where, *TP* is a true positive, meaning the result is properly extracted, *FP* is a false positive, meaning the result is not extracted correctly and *FN* is a false negative, meaning the result is not identified at all.

For evaluation, 195 open-access articles were used in total and were randomly selected on the condition that they contain the element which is being evaluated; for example, yield-strength table-evaluation articles contain tables of yield-strength information. This set was divided into four, such that 100 articles were used to evaluate the extraction of yield-strength information, with 50 evaluating table records and 50 evaluating text records; a different set of 100 articles was used for grain-size information, 45 for table and 50 for text. A manually extracted database was produced for both properties from each respective set of articles. The entries in the manually extracted databases are compared to the automatically extracted ones; when an entry is exactly the same in both databases, it is identified as a true positive; if the record has missing/incorrect information, it is identified as a false positive; false negatives are the entries in the manually extracted database that are missing from the automatically extracted database.

The evaluation metrics of the final databases are shown in Tables 2 and 3. Considering the overall precision, both properties have a precision of around 80%. The difference in precision observed between the table and text parsers is expected since tables are much more structured than free-flowing text and all the necessary information for extraction is usually contained in one table. The main cause of error is the incorrect extraction of the compound name for both property models. Common examples include cases where a trade name is used but it has not been defined in our dictionary and instead some chemical that is mentioned in text is assigned as the "most suitable" candidate given according to the CNER rules. For example, JDBM is a magnesium-based alloy which was not initially written in our dictionary of trade names such that ChemDataExtractor would assign the correctly extracted properties to “magnesium”, which is incorrect. A method to resolve this issue is to add the trade name into the dictionary and re-process the article. However, given that there is a continuously growing number of trade names, it would be impossible to manually construct a complete dictionary of all trade names used. Other error cases include studies where additions of elements to an alloy are discussed. For example, ZK60 is a magnesium-based alloy which has been used in studies where the effect of Scandium additions to the mechanical and structural properties are observed. In these cases, the system would correctly identify the yield strength and grain size but may assign them to either Scandium or ZK60 rather than the full compound name, for example ZK60 + 0.6Sc. Due to the lack of uniformity in writing compounds of this form, it is difficult to define parsing rules that encompass all the possible written forms without the rules being too lenient such that the parser would extract many false positives.

**Table 2:** Yield-strength evaluation metrics.

Model	Precision	Recall	F-score
Yield Strength Text	79.4%	73.6%	76.4%
Yield Strength Table	87.4%	91.9%	89.6%
Overall	83.4%	82.8%	83.0%

**Table 3:** Grain-size evaluation metrics.

Model	Precision	Recall	F-score
Grain Size Text	81.2%	69.6%	77.9%
Grain Size Table	83.6%	82.0%	82.8%
Overall	82.4%	75.8%	78.0.8%

The other common form of error is the incorrect extraction of values. This is much more common in the yield-strength textual evaluation and would occur when multiple stress-related quantities are discussed within the same sentence. For example, Ultimate Tensile Strength is another material property that is often measured alongside yield strength and it is often reported within the same sentence. To illustrate, a common sentence structure that leads to an error is: *"<MATERIAL> has UTS and YS of 150 MPA and 100 MPA."*. According to the parsing rules, the specifier, compound name, value and units are all present in the sentence, and therefore, they will all be extracted. However, the value reported for ultimate tensile strength would often be included in the extraction of yield strength, leading to a false positive in the evaluation. The same reasoning can explain the error in extracting units, although these were rare in the evaluation. While the parsing rules fail in these niche cases, they are able to extract information efficiently at a high precision; they are therefore suitable to be implemented.

The overall recall for both models is also close to 80%, meaning that only a fifth of the available data are not extracted. Recall is often considered to be less important than precision, as long as plenty of source data are available for extraction, since it is better to have correct values than many incorrect values. Accordingly, an argument can be made to make the parsing rules more strict so as to increase precision at the expense of recall if necessary. However, both precision and recall are already high such that the current evaluation metrics of both databases can be considered to be already optimal and are ready to use in data-driven research.

### Distribution of data

**Fig. 2:**
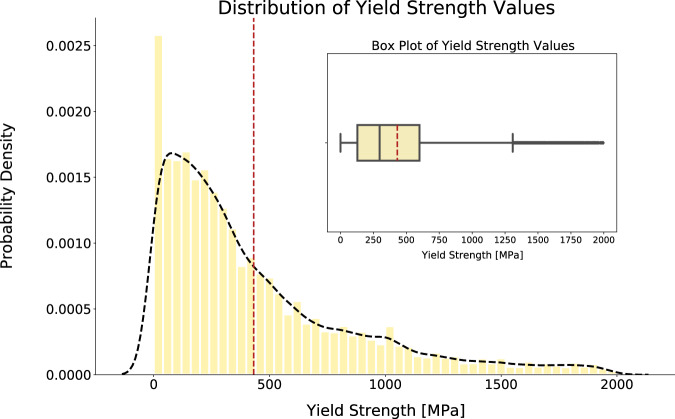
Distribution of yield-strength values. For clarity, values above 2000 MPa have not been plotted. The interquartile range of the data is 546 MPa and values greater than 1500 MPa are considered to statistical extremes which are defined as being 1.5 times the interquartile range above the upper 75% quartile. The dotted red line indicates the mean yield strength at 513.1 MPa. The dotted black line outlines the Kernel Density Estimation of the probability density function. (inset) Shows the box plot of the same distribution.

**Fig. 3:**
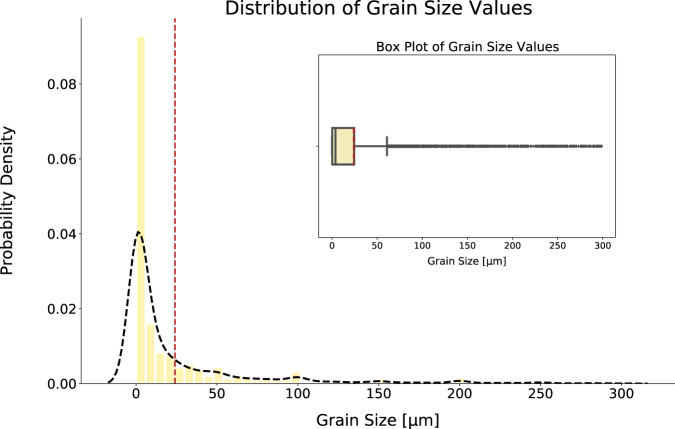
Distribution of grain size values. For clarity, values above 300 *μm* have not been plotted. The interquartile range of the data is 38 *μm* and values greater than 98 *μm* are considered to be statistical extremes which are defined as being 1.5 times the interquartile range above the upper 75% quartile. The dotted red line indicates the mean grain size at 24.02 *μm*. The dotted black line outlines the Kernel Density Estimation of the probability density function. (inset) Shows the box plot of the same distribution.

The distributions of yield-strength and grain-size values are shown in Figs. 2 and 3. The overall shape of the yield-strength distribution matches expectation whereby a positive skew is observed with the majority of values being under 1000 MPa. As yield strength is primarily used in a traditional engineering context dealing with metals and alloys, the typical application area tends to require hard materials with yield strengths ranging between 100 MPa and 1000 MPa. The statistical extreme of the data distribution include any value greater than 1500 MPa, matching the expectation of the typical application area. However, the data-extraction process in this study does not restrict articles to those of metals and alloys when downloading them such that the corpus will most definitely contain papers from other engineering domains as well, such as soft materials and biomedical materials. The materials in these newer engineering domains have yield strengths below 100 MPa; for example, High Density Polyethylene is a common plastic that is studied and has a small measured yield strength of 23 MPa to 29.5 MPa^18^. As the range 0 MPa to 100 MPa is much smaller than 100 MPa to 1000 MPa, the extracted values will be more contained in the lower pressure region, as is reflected by the larger probability density of the lowest-pressure binned data illustrated in Fig. 2. Very large values of yield strengths are also reported; for example, the largest value in the database is 2500 GPa. This is due to an incorrect association of units when multiple properties with units of stress are discussed. These reasons motivate a specialised "Engineering-Ready" yield strength database where the values are restricted to be between 100 MPa and 1500 MPa for the purpose of studying hard materials with more confidence in the precision.

Studies on grain size, in context with yield strength, tend to focus on grain-size refinement, whereby the grain size of a material is reduced to increase yield strength. This is reflected in the positive skew of the grain distribution. The largest bin in this plotted distribution is close to 0 *μm*. While there are novel techniques which allow for such small grains, the extracted values in the nm range tend to be incorrect owing to the ambiguity in language when describing certain manufacturing processes, namely powder metallurgy. Here, grain size refers to something with a definition that is closer to particle size; yet, the same specifier, *"grain size"*, will be used in the text, meaning that the sentence will match the defined parsing rules and be extracted as grain size. Nevertheless, this ambiguity issue affects an extremely small proportion of the data set, where less than 3% of values are below 10 nm and less than 0.5% of values are below 1 nm. In fact, the contribution to this bin is mostly from grain sizes that range between 1 *μm* and 0.1 *μm*. This range is common in grain-size studies, particularly when discussing Ultra Fine Grained materials. Considering the larger grain-size values in the database, values of over 79.3 *μm* are considered to be the statistical extremes. Such grain sizes are not unreasonable, particularly when discussing materials that have had little to no additional processing to reduce the grains. However, very large extracted values seem to represent incorrectly extracted information, most likely due to incorrect unit extraction which causes a difference of multiple orders-of-magnitude between the extracted and reported grain size. A common example of this problem would be when the physical shape of the material is discussed alongside the specifier of grain size. To illustrate, the sentence; *“ the grain size of the 10 mm <MATERIAL> rod is shown in figure*…*”*. The parsing rules will incorrectly associate the grain size to the length of the material. An interesting feature of the grain-size distribution is the way that the peaks of extracted values are at rounded quantities such as 50 *μm* and 100 *μm*. This is due to the tendency of researchers to approximate the grain size when it is not the focus of the study.

### Hall-Petch relationship for AZ31

As a final check on the reliability of the method, the combined database was used to plot a Hall-Petch relationship to compare with results from the literature. The magnesium-based alloy AZ31 was chosen for this as there are many studies on its relationship between grain size and yield strength while it is also being used extensively in real-life applications. Typically, the Hall-Petch relationship states that:

4 $${\sigma }_{y}\propto \frac{1}{\sqrt{d}}$$

where ${\sigma }_{y}$ is the yield strength and *d* is the grain size.

**Fig. 4:**
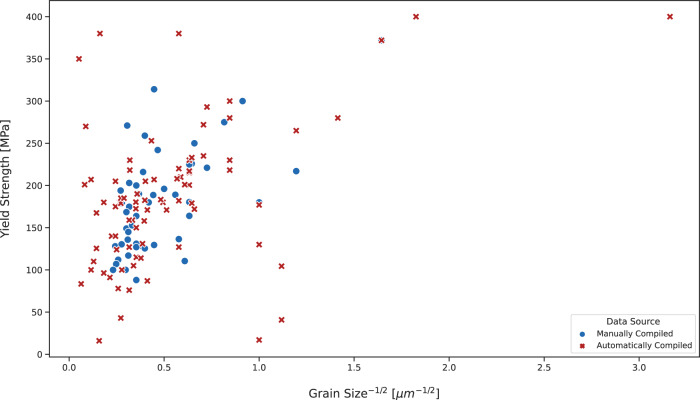
A Hall-Petch relationship plot of AZ31 using (red) automatically extracted data that can be found in our *Combined* database and (blue) manually compiled data from Vinogradov^19^.

A plot of the Hall-Petch relationship of AZ31 using automatically extracted data afforded by this study was compared with a plot that was adapted from manually collected data by Vinogradov *et al*.^19^ as is shown in Fig. 4. Firstly, the similarity between the manually and automatically extracted data distributions can be determined using a two-sample t test for uneven variables. The results of two such statistical tests, one on the yield-strength values and one on the grain-size values, reveal that: for yield-strength values, the t test statistic is −0.433 and the *p* value is 0.666; for grain-size values, the t test statistic is −0.639 and the *p* value is 0.524. None of these values are statistically significant, so the null hypothesis for zero difference between these two distributions is accepted. This means that our automatically generated database provides results that are statistically similar to the results obtained by humans manually extracting data.

When analysing the Hall-Petch relationship in Fig. 4, it is clear that there is no linear relationship and thus, Eq. (4) does not hold. The lack of a clear linear relationship suggests that the Hall-Petch relationship may not be valid for study in the general case whereby data from different studies are used. This suggests that additional factors need to be taken into account and controlled, when studying the exact relationship between yield strength and grain size. Considering our automatically extraxcted data, vertically plotted series of data points in Fig. 4 suggest that it is possible for the same grain size in AZ31 to exhibit different yield strengths, further indicating that other factors play a role in determining the Hall-Petch relationship. Such a conclusion has also been reached by others^19,20^. However, our method is able to determine this conclusion with little human input and using more data to support the arguments made more strongly. For example, using our data, the coefficient of determination can be calculated to give ${R}^{2}=0.21$. This result statistically confirms the unclear Hall-Petch relationship, in the general case, for AZ31. Again, the similarity between the manually curated and autogenerated distributions highlights the efficacy of our method and the potential for it to be implemented in other studies, with confidence that close-to-human-level results will be obtained.

Although similar conclusions have been reached by others^19,20^, the great benefit of our method is that it uses automatically extracted data. This greatly reduces the time required to study a relation, and by achieving results close to what a human would achieve, the database method can be extended to search for the Hall-Petch relationship of many different materials with little human effort. Moreover, applying the same method to automatically extract more properties would permit the study of other relations or even uncover new ones which are hidden in the literature; all without the seemingly never-ending task, for a human, of manually recording results from articles.

## Usage Notes

The databases are available in JSON, CSV and MongoDB BSON formats. These formats are widely supported by most modern programming languages including Python, R and MATLAB which are commonly used in data-driven studies. For lookup purposes, CSV and MongoDB BSON formats allow for efficient querying of data. CSV can be directly imported into popular spreadsheet software while MongoDB enables handling of large databases with more ease. Documentation on how to use MongoDB databases can be found at https://docs.mongodb.com/manual/core/document/. Alternatively, numerous libraries are available that wrap MongoDB functionality into other programming languages, for example the *pymongo* library available in Python.

## Data Availability

The code used to generate the four databases can be found at https://github.com/gh-PankajKumar/ChemDataExtractorStressEng. This repository contains the modified ChemDataExtractor 2.0, webscraping scripts and post-processing tools. The repository contains *propertyExtractor.py*, which was used to automatically extract yield strength and grain size and serves as an example as to how the herein modified version of ChemDataExtractor can be used for extracting engineering-material properties. Also, *propertyExtract_Example.ipynb* is an iPython notebook that walks through the basic steps to extract records from an input article. A static version of the repository is available to download from Figshare^17^

## References

[1] Callister, W. & Rethwisch, D. *Materials Science and Engineering: An Introduction*, 9th Edition: Ninth Edition (John Wiley and Sons, Incorporated, 2013).

[2] Hey, T., S.K.*et al*. *The fourth paradigm: data-intensive scientific discovery*, vol. 1 (Microsoft research Redmond, WA, 2009).

[3] Agrawal A, Choudhary A (2016). Perspective: Materials informatics and big data: Realization of the "fourth paradigm" of science in materials science. APL Materials.

[4] de Pablo JJ, Jones B, Kovacs CL, Ozolins V, Ramirez AP (2014). The materials genome initiative, the interplay of experiment, theory and computation. Current Opinion in Solid State and Materials Science.

[5] Jain A (2013). The Materials Project: A materials genome approach to accelerating materials innovation. APL Materials.

[6] O’Mara J, Meredig B, Michel K (2016). Materials data infrastructure: a case study of the citrination platform to examine data import, storage, and access. JOM.

[7] Blaiszik B (2016). The materials data facility: Data services to advance materials science research. JOM.

[8] White AA (2013). Big data are shaping the future of materials science. MRS Bulletin.

[9] Krallinger M, Rabal O, Lourenço A, Oyarzabal J, Valencia A (2017). Information retrieval and text mining technologies for chemistry. Chemical Reviews.

[10] Eltyeb S, Salim N (2014). Chemical named entities recognition: a review on approaches and applications. Journal of Cheminformatics.

[11] Hall EO (1951). The deformation and ageing of mild steel: III discussion of results. Proceedings of the Physical Society. Section B.

[12] Petch N (1953). The cleavage strength of polycrystals. Journal of the Iron and Steel Institute.

[13] Swain MC, Cole JM (2016). Chemdataextractor: a toolkit for automated extraction of chemical information from the scientific literature. Journal of chemical information and modeling.

[14] Mavracic, J., Court, C. J., Isazawa, T., Elliott, S. R. & Cole, J. M. Chemdataextractor 2.0: Auto-populated ontologies for materials science. *J. Chem. Inf. Model*. (2021 (submitted)). 10.1021/acs.jcim.1c00446 34529432

[15] Feldman, R. & Sanger, J. Information Extraction, 94–130. *The Text Mining Handbook* (Cambridge University Press, nil).

[16] Kononova O (2019). Text-mined dataset of inorganic materials synthesis recipes. Scientific Data.

[17] Kumar P, Cole JM, Kabra S (2021). figshare.

[18] Polymer database: High-density polyethylene (hdpe). Chemical Retrieval on the Web https://www.polymerdatabase.com/Commercial%20Polymers/HDPE.html.

[19] Vinogradov A, Serebryany VN, Dobatkin SV (2017). Tailoring microstructure and properties of fine grained magnesium alloys by severe plastic deformation. Advanced Engineering Materials.

[20] Vinogradov A (2017). Effect of severe plastic deformation on tensile and fatigue properties of fine-grained magnesium alloy zk60. Journal of Materials Research.

